# The detrimental effect of iron on OA chondrocytes: Importance of pro‐inflammatory cytokines induced iron influx and oxidative stress

**DOI:** 10.1111/jcmm.16581

**Published:** 2021-05-03

**Authors:** Xingzhi Jing, Ting Du, Tao Li, Xiaoxia Yang, Guodong Wang, Xiaoyang Liu, Zhensong Jiang, Xingang Cui

**Affiliations:** ^1^ Department of spine surgery Shandong Provincial Hospital affiliated to Shandong First Medical University Jinan China; ^2^ Department of Otolaryngology–Head and Neck Surgery Shandong Provincial ENT Hospital Affiliated to Shandong University Jinan China; ^3^ Department of spine surgery Shandong Provincial Hospital Cheeloo College of Medicine Shandong University Jinan China

**Keywords:** iron overload, mitochondrial dysfunction, osteoarthritis, oxidative stress, pro‐inflammatory cytokines

## Abstract

Iron overload is common in elderly people which is implicated in the disease progression of osteoarthritis (OA), however, how iron homeostasis is regulated during the onset and progression of OA and how it contributes to the pathological transition of articular chondrocytes remain unknown. In the present study, we developed an in vitro approach to investigate the roles of iron homeostasis and iron overload mediated oxidative stress in chondrocytes under an inflammatory environment. We found that pro‐inflammatory cytokines could disrupt chondrocytes iron homeostasis via upregulating iron influx transporter TfR1 and downregulating iron efflux transporter FPN, thus leading to chondrocytes iron overload. Iron overload would promote the expression of chondrocytes catabolic markers, MMP3 and MMP13 expression. In addition, we found that oxidative stress and mitochondrial dysfunction played important roles in iron overload‐induced cartilage degeneration, reducing iron concentration using iron chelator or antioxidant drugs could inhibit iron overload‐induced OA‐related catabolic markers and mitochondrial dysfunction. Our results suggest that pro‐inflammatory cytokines could disrupt chondrocytes iron homeostasis and promote iron influx, iron overload‐induced oxidative stress and mitochondrial dysfunction play important roles in iron overload‐induced cartilage degeneration.

## 1 INTRODUCTION

Osteoarthritis (OA) is the most frequent adult joint disease which is characterized by synovial tissue inflammation and progressive loss of joint articular cartilage. Chondrocytes are the only cell type in cartilage responsible for the production of extracellular cartilage matrix including type II collagens and aggrecan, which play important roles in maintaining cartilage function. Although age, mechanical overload, joint injuries and gender are major risk factors for the disease, the intrinsic physiological and molecular mechanisms of OA still remain poorly understood.^1^

Iron overload is common in tissues of elderly people and has been implicated to be responsible for many diseases or pathological conditions.^2^ Abnormal iron metabolism has recently been implicated in the disease progression of OA. Multiple independent clinical studies on OA in the elderly people found that people with high serum ferritin levels have a 4‐fold increased risk of OA, and that serum ferritin levels were positively correlated with radiographic severity.^3^ OA is a common complication of many diseases with abnormal cartilage iron overload, including haemophilic arthropathy, hereditary hemochromatosis and rheumatoid arthritis.^4^ Iron deposition in joint synovium and cartilage is an important pathological process in OA development, elevated iron concentration in synovial fluid and iron crystal deposition was observed in osteoarthritis and traumatic cartilage damage.^5^ Hereditary hemochromatosis (HH) is a chronic systemic iron overload disease that is caused by mutations in the HFE gene. Recently, Camacho A et al demonstrated that HFE‐KO mice developed more severe knee OA than wild‐type (wt) mice after joint destabilization induced by partial meniscectomy.^6^ However, the underlying mechanism of iron takes parts in OA development remains unknown.

Iron plays important roles in normal cellular processes including oxygen storage and transport, energy metabolism and DNA, RNA synthesize. Delicate regulation of iron homeostasis is required for maintaining normal cellular function, while excessive iron would damage cells by the increasing production of reactive oxygen species (ROS). Because of Fenton reaction, excess iron could generate highly toxic hydroxyl radicals and result in the peroxidation of membrane lipids, mitochondrial dysfunction, cellular proteins and nucleus acids damage and ultimately ferroptosis.^7^ Oxidative stress and mitochondrial dysfunction were demonstrated to be an important contributor to joint OA development.^8^, ^9^ Mitochondria is reported to be the main cellular organ for iron metabolism and ROS production. Iron could participate in mitochondrial oxidative respiratory chain by exchanging a single electron with a number of substrates which can lead to the generation of ROS.^10^ Increased ROS production could damage mitochondria as well as other structures, leading to cell death. Damaged mitochondria could in turn produce excess ROS and result in decreased collagen production and increased matrix‐degrading enzyme excretion.^9^ Mitochondrial fission and fusion process could remove damaged mitochondria and restore mitochondrial morphology and function. During fission, Dynamin‐related protein 1 (Drp1) is recruited from the cytosol to the mitochondria to form helixes around mitochondria and sever both the outer mitochondrial membranes (OMM) and inner mitochondrial membranes (IMM) by constriction. Other mitochondrial fission proteins that regulate mitochondrial fission included fission 1 homologue protein (FIS1) and mitochondrial fission factor (MFF). The damaged mitochondria are subsequently degraded by mitophagy. Mitophagy is a mitochondrial autophagy process that selectively removes damaged mitochondria.^11^

Because of its double‐edged sword effect in the body, iron homeostasis is delicately regulated. Cellular iron homeostasis is achieved by modulating the expression of proteins involved in iron uptake, storage, and export. Iron regulator proteins (IRPs) sense the concentration of active iron in the mitochondria and regulate the expression of iron uptake associated proteins transferrin receptor 1(TfR1) and divalent metal transporter 1 (DMT1).^12^ IRP could also bind to the IRE of the 5' untranslated region of ferroportin (FPN) and inhibit the iron release from the cell.^13^ Disruption of iron homeostasis is reported to take parts in many diseases, such as Parkinson disease, Alzheimer disease, cardiovascular diseases and osteoporosis.^14^ Recent researches indicated that many risk factors, including ageing, mechanical overload, inflammation would disrupt cellular homeostasis and thus lead to iron overload.^15^

The association between iron overload and OA pathogenesis is broadly appreciated regarding its effect on ROS production and oxidative stress.^16^ OA is characterized by synovial tissue inflammation and cartilage degeneration. However, how iron homeostasis is regulated in chondrocytes under an inflammatory environment and how it contributes to the pathological transition of articular chondrocytes remain unknown. In the present study, we developed an in vitro approach using pro‐inflammatory cytokines to mimic OA pathological conditions. We sought to investigate how iron homeostasis is regulated in chondrocytes under an inflammatory environment, and the roles of iron overload and iron overload mediated oxidative stress in cartilage degeneration.

## 2 MATERIALS AND METHODS

### 2.1 Cell isolation and culture

Chondrocytes were obtained from five days old C57/BL6 male mice. Briefly, bilateral knee joints were isolated and cartilage was minced into small pieces. After washed by cold PBS, cartilage pieces were digested with 0.25% trypsin‐EDTA for 30 minutes at 37°C. Then trypsin‐EDTA was removed and sample pieces were washed by PBS twice. Samples were then re‐suspended in 0.25% collagenase solution at 37°C for 6 hours. Finally, cells were cultured in DMEM/F12 medium containing 10% FBS and passaged when cells reached 80% confluence. Chondrocytes at passage 1 and 2 were used in our study. All animal protocols were approved by the Institutional Animal Care of the Shandong Provincial Hospital affiliated to Shandong First Medical University.

### 2.2 Western blot analysis

Briefly, chondrocytes were homogenized on ice and lysed with RIPA lysis buffer (Boster, Wuhan, China). After 30 minutes, the lysates were collected and centrifugated at 12 800 *g* for 20 minutes at 4℃. 25 μg proteins from each sample were separated by 10% SDS‐PAGE gel and then transferred to polyvinylidine difluoride PVDF membranes (pore size 0.45 μmol/L, Millipore, Billerica, MA), which were subsequently incubated with targeted primary antibodies anti MMP3 (17873‐1‐AP, proteintech, USA), MMP13 (ab39012, Abcam, USA), FIS1 (10956‐1‐AP, proteintech, USA), DRP1 (#8570, CST, USA), MFF (#84580, CST, USA), iron regulatory protein 1 (IRP1, ab126595, Abcam, USA), iron regulatory protein 2 (IRP2, ab110321, Abcam, USA), transferrin receptor (TfR1, ab84036, Abcam, USA), Ferroportin (FPN, ab78066, Abcam, USA), β‐actin (#BM0627, Boster, Wuhan) at 4°C overnight. After being washed for three times with TBST, membranes were incubated with respective secondary antibodies at room temperature for 1 hour. Bands and band density were detected using Western ECL Substrate Kit (Thermo Pierce, USA) and analyzed with the built‐in software of Bio‐Rad scanner (Bio‐Rad, Hercules, CA). Images were acquired with Bio‐Rad scanner (Hercules, CA) and densitometry was quantified by digital image analysis software (Quantity One, Bio‐Rad, Hercules, CA).

### 2.3 Reverse transcription and real‐time polymerase chain reaction (RT‐PCR) analysis

Total RNA isolation and quantitative RT‐PCR amplification were performed as previously described.^17^ Briefly, complementary DNA (cDNA) synthesis was performed by using a first Strand cDNA Synthesis Kit (Toyobo, Ōsaka, Japan). cDNA was amplified using specific primers: ADAMTS5, Forward: 5′‐TATGACAAGTGCGGAGTATG‐3′, Reverse: 5′‐TTCAGGGCTAAATAGGCAGT‐3′; MMP3, Forward: 5′‐ATGCCCACTTTGATGATGATGAAC‐3′, Reverse: 5′‐CCACGCCTGAAGGAAGAGATG‐3′; MMP13, Forward: 5′‐GCTGGACTCCCTGTTG‐3′, Reverse: 5′‐TCGGAGCCTGTCAACT‐3′; GAPDH: Forward: 5′‐CTCCCACTCTTCCACCTTCG‐3′, Reverse: 5′‐TTGCTGTAGCCGTATTCATT‐3′;

### 2.4 Measurement of intracellular iron levels

Iron‐sensitive fluorescent Calcein‐AM dye was used to evaluate the intracellular iron levels. Briefly, cells were pre‐treated with 10 ng/mL IL‐1β for 24 hours and then incubated with 0.5 μmol/L Calcein‐AM for 30 minutes at 37°C. Chondrocytes were then washed with PBS for three times to wash out the excess calcein dye. 100 μmol/L FAC was added into medium and ferrous iron influx into chondrocytes was determined 15 minutes later by the quenching of metal‐bound Calcein‐AM. Chondrocytes were imaged using a fluorescence microscope (Evos fl auto; Life Technologies, Carlsbad, CA) at an excitation wavelength of 488 nm and an emission wavelength of 575 nm. To quantify the fluorescence values, four separate fields under a microscope at ×200 magnification were randomly selected and the mean fluorescence signal of 25‐30 single cells was recorded with Image Pro Plus.

### 2.5 Evaluation of intracellular ROS

Chondrocytes were treated with FAC (ferric ammonium citrate) with or without 100 μmol/L DFO (deferoxamine) or NAC (N‐acetyl‐L‐cysteine) for 24 hours. After treatment, the intracellular ROS level determination was performed using the Reactive Oxygen Species Assay Kit (S0033, Beyotime, China) as previously described.^18^ To quantify intracellular ROS level, chondrocytes were collected and the mean fluorescence intensity was evaluated with a FACS Calibur flow cytometer (BD Biosciences, Franklin Lakes, NJ).

### 2.6 Measurement of mitochondrial membrane potential

Chondrocytes were cultured in 24‐well plate and FAC (100 μmol/L) with or without 100 μmol/L DFO or NAC for 24 hours. Then cells were washed with DMEM‐F12 and incubated with JC‐1 staining solution. After incubated for 20 minutes at 37℃ in the darkness, cells were washed twice with JC‐1 washing buffer. The fluorescence was observed using a fluorescence microscope. To quantify mitochondrial membrane potential in chondrocytes treated with FAC with/without NAC (100 μmol/L), an antioxidant or DFO (100 μmol/L), an iron chelator that could reduce cellular iron content, cells were collected and the ratio of red fluorescence intensity to green fluorescence intensity was evaluated with a FACS Calibur flow cytometer (BD Biosciences).

### 2.7 Mitochondrial specific fluorescence staining

The morphological changes of mitochondria were assessed using Mito‐Tracker Green (Beyotime Biotechnology, Shanghai, China). Briefly, chondrocytes were treated with FAC (100 μmol/L) and then washed with DMEM/F12. After incubated with diluted Mito‐Tracker Green solution at 37℃ for 30 minutes in the darkness. The mitochondrial morphology was imaged under a fluorescence microscope (Evos fl auto; Life Technologies, Carlsbad, CA).

### 2.8 Evaluation of apoptosis by Annexin V‐FITC/PI staining

Annexin V‐FITC/PI kit (Abbkine, California, USA) was used to investigate the apoptotic effect of FAC. Chondrocytes were treated with FAC (100 μmol/L) with or without 100 μmol/L DFO or NAC for 24 hours. Cells were then collected and washed three times with PBS. Then chondrocytes were incubated with Annexin V‐FITC at 4℃ for 15 minutes in the darkness. PI (propidium iodide) was added 15 minutes before evaluation using a FACS Calibur flow cytometer (BD Biosciences, Franklin Lakes, NJ).

### 2.9 Statistical analysis

The comparisons between multiple groups, such as RT‐PCR, western blot and immunofluorescence analyses were performed using multiple comparisons by one‐way ANOVA followed by Tukey's test. For western blot and intracellular ROS evaluation data, Student's *t* test and one‐way ANOVA with Dunnett's test were used for pairwise comparisons and multi‐group comparison, respectively. Results are represented as mean ± SD, *P* values <.05 were considered to be significant. All analyses were performed with GraphPad Prism software (Version 6.0).

## 3 RESULTS

### 3.1 The pro‐inflammatory cytokines disrupted iron homeostasis with increased TfR1 expression and decreased FPN expression

**FIGURE 1:**
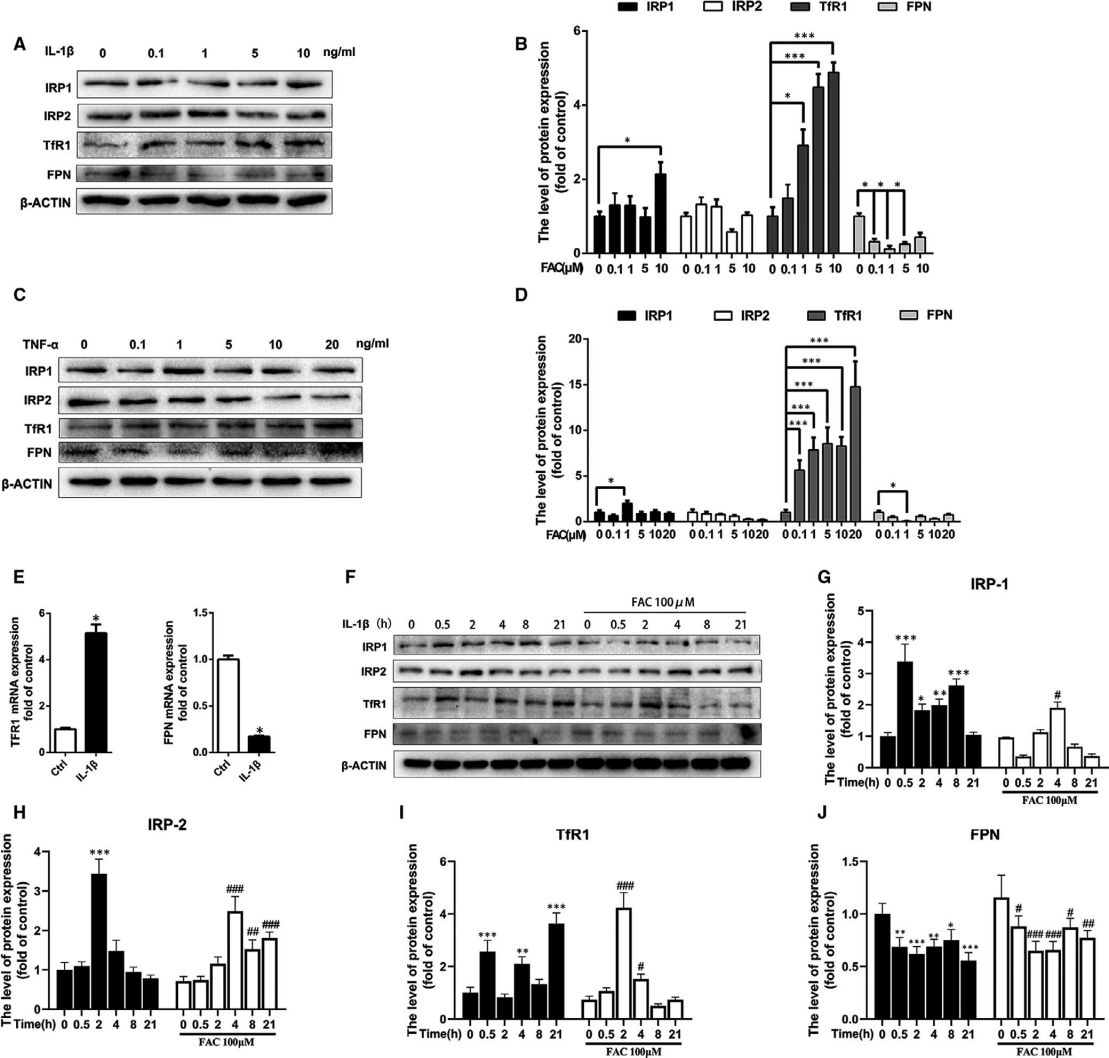
Pro‐inflammatory cytokines IL‐1β and TNF‐α disrupted iron homeostasis in chondrocytes via upregulating iron influx protein, downregulating iron efflux protein. A‐D, Chondrocytes were treated with increasing concentrations of IL‐1β or TNF‐α and iron regulators, IRP1/2, TfR1 and FPN proteins expression were determined by western blot analysis. The band density of IRP1/2, TfR1 and FPN were quantified and normalized to control. E, Chondrocytes were treated with 10 ng/mL IL‐1β for 24 h and RT‐PCR was conducted to evaluated TfR1 and FPN gene expression. F, Chondrocytes were treated with 10 ng/mL IL‐1β with or without 100 μmol/L FAC for different time intervals and iron regulators, IRP1/2, TfR1 and FPN protein expression were determined by western blot analysis. G‐J, The band density of IRP1/2, TfR1 and FPN were quantified and normalized to control. Data are presented as mean ± SD from three different experiments. *P < .05; **P < .01; ***P < .001 vs Ctrl; ^#^ *P* < .05; ^##^ *P* < .01; ^###^ *P* < .001vs FAC (100 μmol/L, 0 min)

To elucidate the change of iron homeostasis and the associated iron regulators in chondrocytes under an inflammatory environment, chondrocytes were treated with IL‐1β and TNF‐α and western blot analysis was performed to detect the protein levels of IRP1/2, TfR1 and FPN. IL‐1β and TNF‐α showed a similar trend that IRP1 and TfR1 were upregulated and FPN was downregulated after treatment of various concentrations of IL‐1β and TNF‐α. While no significant difference was observed in IRP2 protein expression after both IL‐1β and TNF‐α treatment (Figure 1A‐D). RT‐PCR experiment obtained similar results that IL‐1β significantly promoted iron influx mediators TfR1, inhibited iron efflux mediator FPN genes expression (Figure 1E). To further determine the iron regulator proteins expression induced by pro‐inflammatory cytokines, primary chondrocytes were treated with 10 ng/mL IL‐1β with or without 100 μmol/L FAC for different time intervals and western blot assay was conducted to investigate the expression of IRP1/2, TfR1 and FPN. Our results indicated that IL‐1β promoted TfR1 expression, inhibited FPN expression. IRP1 protein expression increased after 30 minutes of IL‐1β stimulation. While IRP2 protein increased after 2 hours of IL‐1β stimulation and returned to normal after 4 hours of IL‐1β stimulation. 100 μmol/L FAC co‐treatment inhibited IRP1 and TfR1 protein expression and promoted FPN protein expression (Figure 1F‐J).

### 3.2 Iron overload promoted expression of chondrocyte matrix‐degrading enzymes

**FIGURE 2:**
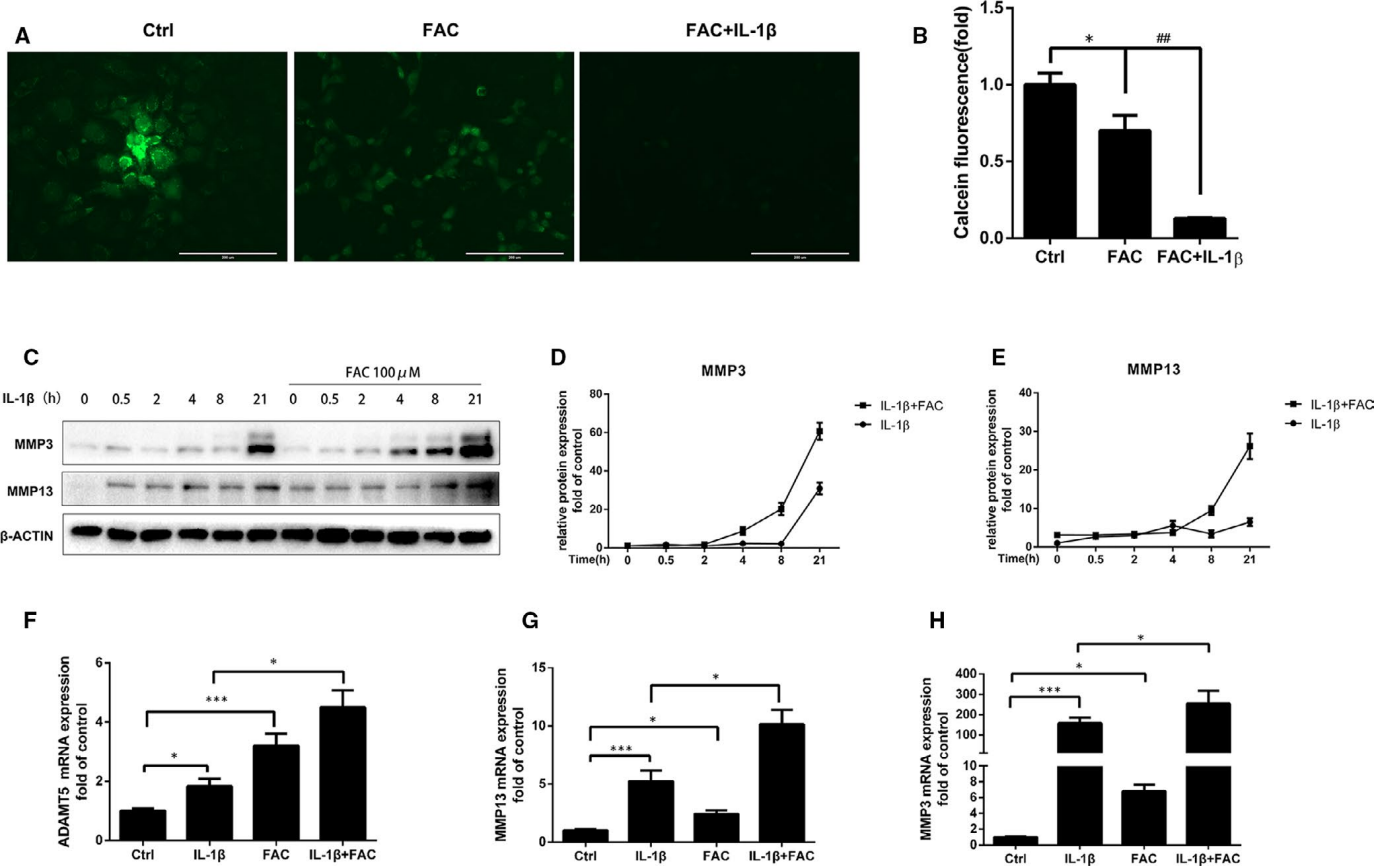
Elevated iron influx induced by IL‐1β promoted chondrocytes matrix‐degrading proteins (MMP3, MMP13) expression. A‐B, Chondrocytes were treated with 100 μmol/L FAC in the presence or absence of 10 ng/mL IL‐1β. The quenching of green fluorescence was observed by confocal microscopy at 15 min after treatment. Scale Bars = 200 μmol/L. *P < .05 vs Ctrl group; ^##^ *P* < .01 vs FAC treatment group. C‐E, Chondrocytes were treated with 10 ng/mL IL‐1β with or without 100 μmol/L FAC for different time intervals and MMP3, MMP13 proteins expression were determined by western blot analysis. The band density of MMP3, MMP13 were quantified and normalized to control. F‐H, Chondrocytes were treated with 10 ng/mL IL‐1β with or without 100 μmol/L FAC for 24 h and RT‐PCR analysis was conducted to examine ADAMTS5, MMP3 and MMP13 gene expression. Data are presented as mean ± SD from three different experiments; *P < .05; ***P < .001

To evaluate the detrimental effect of iron in chondrocytes, primary mouse chondrocytes were initially treated with 100 μmol/L FAC with or without 10 ng/mL IL‐1β, fluorescence dye calcein was used to assess the ferrous iron uptake process in chondrocytes. As shown in Figure 2A‐B, the fluorescence intensity decreased after FAC treatment, indicating elevated iron concentration in chondrocytes. While the fluorescence intensity was more significantly decreased with IL‐1β co‐treatment, indicating that IL‐1β promoted chondrocytes iron uptake. To evaluate the detrimental effect of iron influx in chondrocytes, chondrocytes were treated with 10 ng/mL IL‐1β for different time intervals with or without 100 μmol/L FAC. As shown in Figure 2C‐E, obvious overproduction of MMP3 and MMP13 were observed after 21 hours IL‐1β treatment, while FAC accelerated the production of MMPs. Significant overproduction of MMP3 and MMP13 were observed after 4 hours of IL‐1β and FAC co‐treatment. Similar results were observed in RT‐PCR assay. FAC co‐treatment could further promote matrix‐degrading enzymes such as MMPs and ADAMTS5 expression which were treated with IL‐1β for 24 hours (Figure 2F‐H).

### 3.3 Iron overload promoted ROS production and impairs mitochondrial dynamics in chondrocytes

**FIGURE 3:**
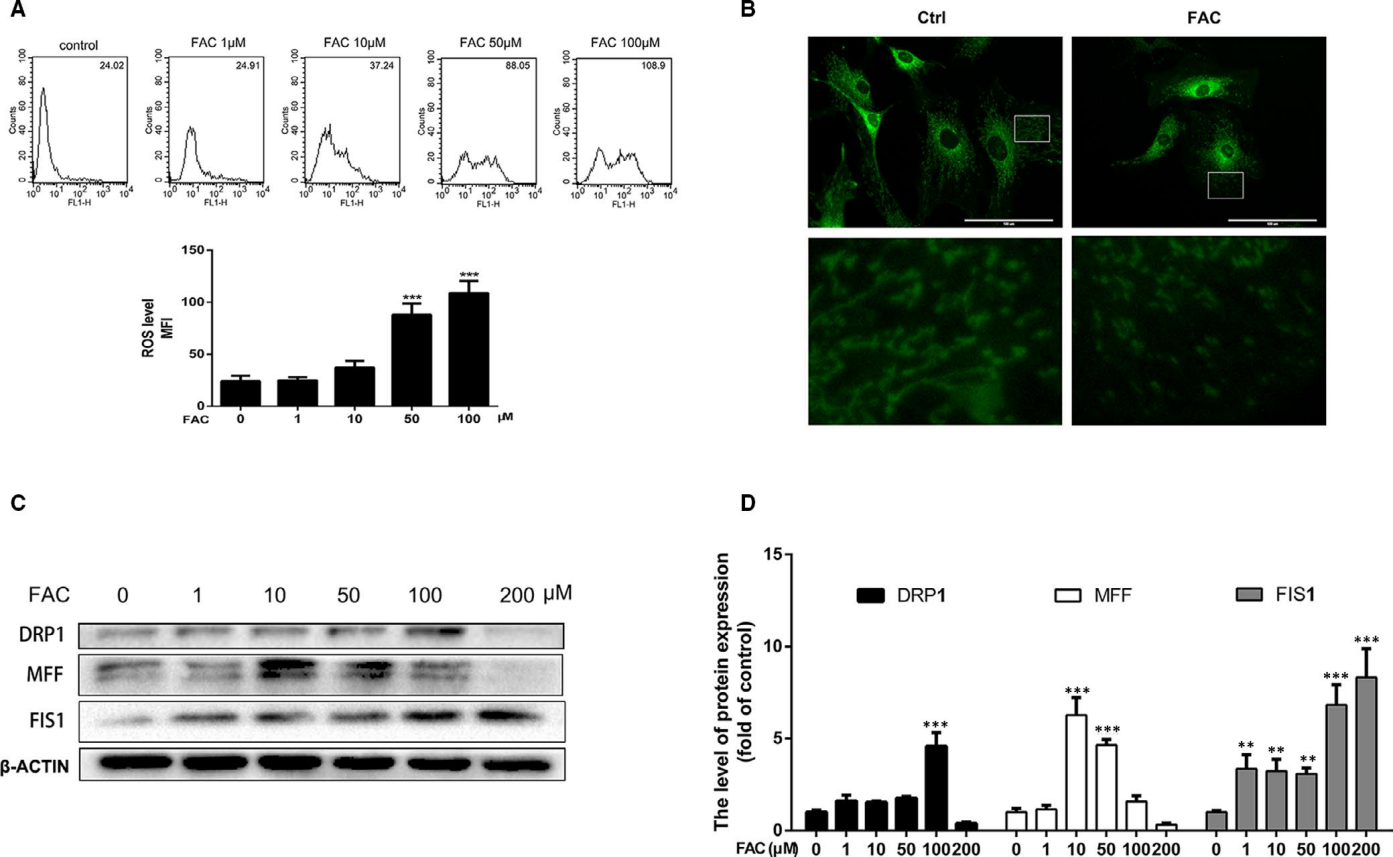
Iron overload promoted ROS production and impaired mitochondrial dynamics in chondrocytes. A, Chondrocytes were treated with various concentrations of FAC and flow cytometric analysis was conducted to quantify ROS production. MFI (mean fluorescence intensity). B, Representative fluorescence photographs of mitochondria after 100 μmol/L FAC treatment for 24 h. The morphology of the mitochondria is stained by mito‐tracker green. Scale bar = 100 μmol/L. C, Chondrocytes were treated with various concentrations of FAC for 24 h and mitochondrial fission proteins, DRP1, MFF and FIS1 were determined by western blot. D, The band density of DRP1, MFF and FIS1 were quantified and normalized to control. Data are presented as mean ± SD from three different experiments. **P < .01; ***P < .001

To have a better understanding of the underlying mechanisms of iron overload in promoting chondrocytes matrix‐degrading enzymes expression, chondrocytes were treated with different concentrations of FAC for 24 hours and incubated with DCFH‐DA for 30 minutes to evaluate ROS production. Flow cytometric analysis showed that FAC promoted ROS generation in a dose‐dependent manner (Figure 3A). We next detected the effect of iron overload in the morphological dynamics of mitochondria. The mitochondria of healthy chondrocytes exhibited long and wire‐like shape, while after treatment of FAC, the number of shortened mitochondria dramatically increased (Figure 3B). Mitochondrial fission and fusion are known to regulate the function and morphology of mitochondria. A number of studies have shown that mitochondrial fission is an early event during the process of cell death. Our results showed that mitochondrial fission proteins (DRP1, MFF, FIS1) expression were upregulated within 100 μmol/L and downregulated when over 100 μmol/L after FAC treatment for 24 hours (Figure 2C‐D). These results indicate that FAC could promote mitochondrial fission. Overdose and prolonged FAC treatment would inhibit mitochondrial fission and fusion, which might inhibit mitophagy and further cause mitochondrial destruction.

### 3.4 Oxidative stress and mitochondrial dysfunction mediates iron overload‐induced chondrocytes apoptosis and matrix metalloproteinases expression

**FIGURE 4:**
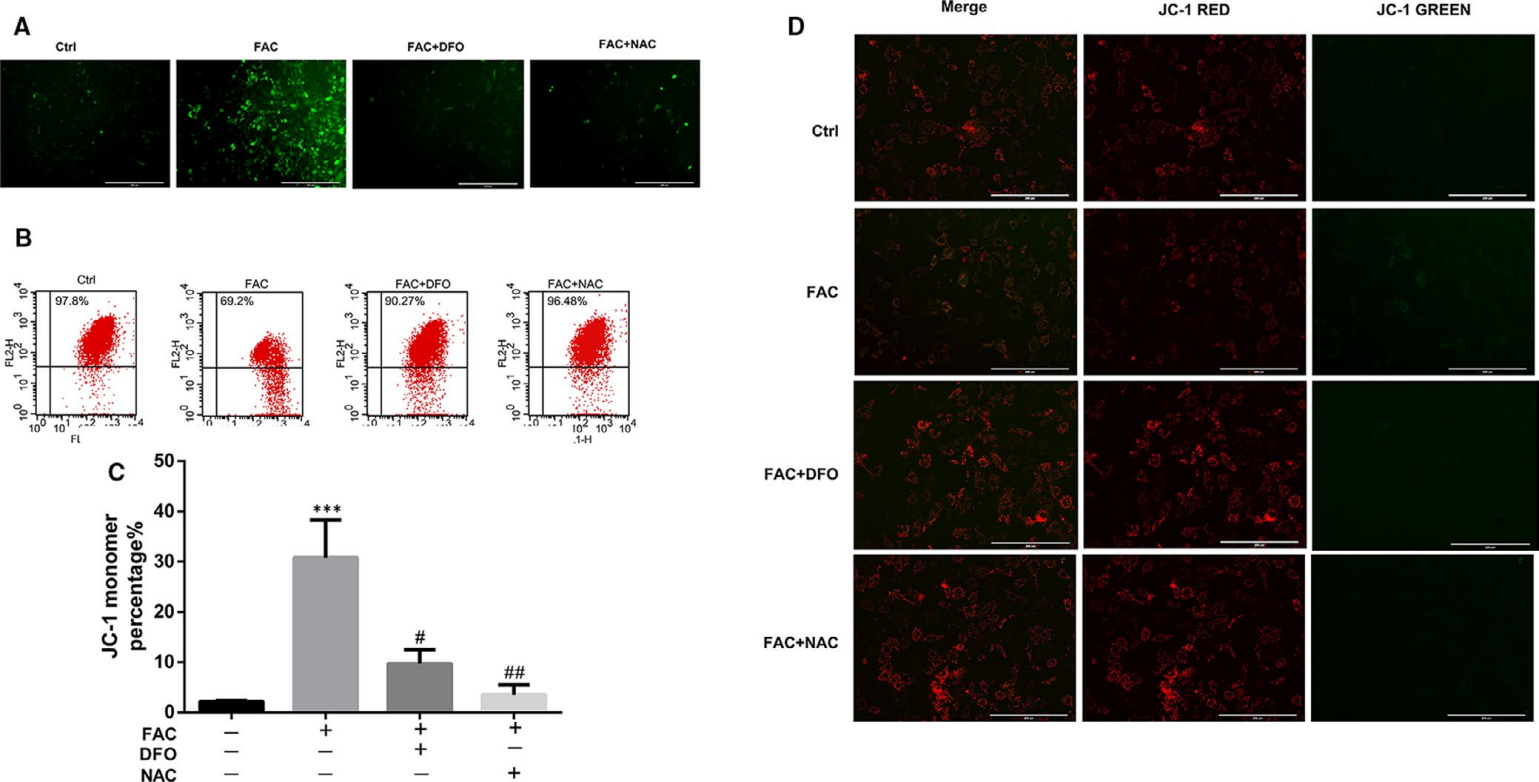
Iron chelator DFO or antioxidant NAC inhibited ROS production and protected against iron overload induced collapse of mitochondrial membrane potential. A, Representative fluorescence photographs of intracellular ROS in chondrocytes. Scale bars = 400 μmol/L. B‐D, Chondrocytes were treated with 100 μmol/L FAC with 100 μmol/L NAC or DFO for 24 h, JC‐1dye immunofluorescence and flow cytometric analysis were conducted to detect the mitochondrial membrane potential. Scale bars = 200 μmol/L. Data are presented as mean ± SD from three different experiments. ***P < .001 vs Ctrl; ^#^ *P* < .05; ^##^ *P* < .01 vs FAC treatment

**FIGURE 5:**
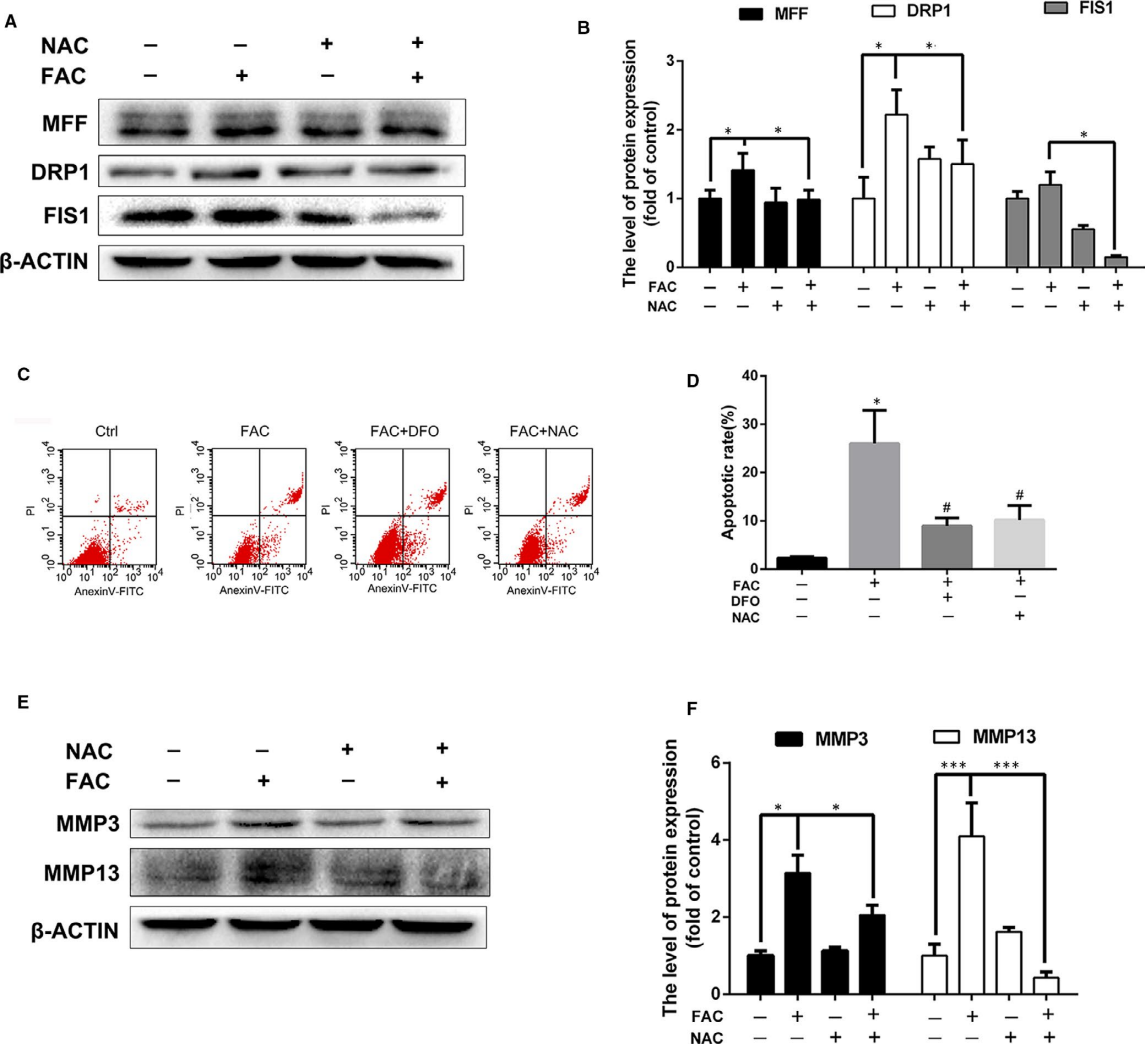
Iron chelator DFO or antioxidant NAC inhibited iron overload induced apoptosis, mitochondrial dysfunction and matrix metalloproteinases (MMPs) upregulation. A‐B, Chondrocytes were treated with 100 μmol/L FAC with or without 100 μmol/L NAC for 24 h and mitochondrial fission protein (DRP1, MFF, FIS1) were detected using western blot analysis. The band density were quantified and normalized to control. C‐D, Chondrocytes were treated with 100 μmol/L FAC with 100 μmol/L NAC or DFO for 24 h, Annexin V‐FITC/PI flow cytometric analysis was conducted to detect the apoptosis rate. Data are presented as mean ± SD. *P < .05 vs Ctrl group; ^#^ *P* < .05 vs FAC treatment group. E‐F, Chondrocytes were treated with 100 μmol/L FAC with or without 100 μmol/L NAC for 24 h and matrix metalloproteinases (MMP3, MMP13) were detected using western blot analysis. The band density were quantified and normalized to control. Data are presented as mean ± SD from three different experiments. *P < .05; ***P < .001

Because of Fenton reaction, excess iron could generate highly toxic hydroxyl radicals and result in mitochondrial dysfunction. We next examined whether excess iron‐induced oxidative stress and mitochondrial dysfunction mediates the accelerated progression of OA. Primary chondrocytes were treated with DFO to chelate iron ions, or NAC to inhibit iron overload‐induced oxidative stress. As shown in Figure 4A, excess iron‐induced ROS overproduction was significantly inhibited by DFO or NAC treatment. Collapse of mitochondrial membrane potential (MMP) represents mitochondrial dysfunction. As shown in Figure 4B‐D, 100 μmol/L FAC treatment significantly increased the green fluorescence intensity of JC‐1 monomers, suggesting that iron overload has a detrimental effect on the mitochondrial function, leading to reduction of MMP. NAC treatment reversed the detrimental effect of iron overload in mitochondrial dysfunction which exhibited a similar effect with DFO. Similar results were observed by western blot assay that excess iron‐induced upregulation of mitochondrial fission proteins, DRP1, MFF and FIS1 which were reversed after DFO or NAC co‐treatment (Figure 5A‐B), indicating iron overload‐induced mitochondrial dysfunction was associated with oxidative stress through ROS generation.

Next, we examined whether NAC or DFO could reverse iron overload‐induced chondrocytes apoptosis and matrix metalloproteinases (MMPs) upregulation. As shown in Figure 5C‐D, FAC at 100 μmol/L significantly promoted chondrocytes apoptosis while NAC or DFO treatment decreased chondrocytes apoptosis rate induced by FAC. A hallmark of OA chondrocytes is the increased production of matrix‐degrading enzymes, such as MMPs. As shown in Figure 5E‐F, FAC‐induced MMP3 and MMP13 upregulation were significantly inhibited by DFO and NAC, indicating that reducing iron concentration using iron chelators or antioxidant drugs are potential approaches to inhibit detrimental effects induced by iron overload.

## 4 DISCUSSION

Osteoarthritis is characterized by overproduction of ROS in afflicted joints.^8^ Iron overload, due to its effect in the production of ROS and crystal deposition in the joints, is implicated in the disease progression of OA.^19^ Recent clinical studies indicated the link between iron overload and the incidence and progression of OA.^20^ However, no evidence available to date clearly shows the involvement of iron homeostasis in the OA pathogenesis. In the present study, we demonstrated that pro‐inflammatory cytokines disrupted chondrocytes iron homeostasis via upregulating iron influx mediators TfR1, downregulating iron efflux mediator FPN expression. Excess iron could promote OA‐related catabolic markers MMP3 and MMP13 expression in chondrocytes. Moreover, we found that oxidative stress and mitochondrial dysfunction play important roles in iron overload‐induced chondrocytes OA‐related catabolic markers expression.

Although many researches and clinical evidences indicated that iron might be involved in the development of OA, there are still many problems that need to be further clarified. Epidemiological evidence showed that iron overload is a common physiological and pathological state in the elderly due to the lack of an effective way to excrete iron in the human body.^21^ It is reported that the serum ferritin concentration in women increased rapidly after menopausal with 106 ng/mL which is more than twice that of premenopausal women. The average serum ferritin concentration of middle‐aged and elderly men is 121 ng/mL, which is 3‐4 times that of adolescent.^22^ But not all elderly people would eventually develop into OA, we guess this may be related to cellular iron metabolism regulation. Iron is a "double‐edged sword", the normal function of cells require the participation of iron ions, while excess iron ions might cause oxidative stress and mitochondrial dysfunction. Therefore, cellular iron homeostasis is delicately regulated by iron metabolism‐related proteins.^23^ A Camacho and M Simão et al have also found that iron overload alone in joints of hereditary hemochromatosis mouse model seems not enough to induce OA, but it can significantly promote OA development after joint destabilization induced by partial meniscectomy.^6^ We speculate that the imbalance of iron metabolism in chondrocytes under pathological conditions may be an important prerequisite for the involvement of iron ions in the development of OA. However, how iron homeostasis is regulated under OA pathogenic conditions remains unknown.

Iron homeostasis is delicately regulated in the human body, but many risk factors, including age, mechanical load, inflammation could disturb iron homeostasis and cause iron accumulation in tissues.^24^ Pro‐inflammatory cytokines, such as interleukin‐1β (IL‐1β) and tumour necrosis factor‐a (TNF‐a), are major components of OA inflammation that play key roles in OA primary cartilage damage.^25^ In the present study, we found that IL‐1β or TNF‐α increased ferrous iron influx and decreased iron efflux, with increased TfR1 expression and decreased FPN expression. What's more, the level of IRP1 was elevated in IL‐1β or TNF‐α treated chondrocytes. IRPs could sense cellular iron status and regulate the iron homeostasis by binding to iron‐responsive element (IRE). Upregulated IRP1 binding to IRE increased TfR1 mRNA stability and repressed FPN mRNA translation.^23^, ^26^ Our results were consistent with previous studies indicating that IRP1 was upregulated after pro‐inflammatory cytokines stimulation in Parkinson's disease.^27^

We next investigated the detrimental effect of iron influx induced by pro‐inflammatory cytokines in chondrocytes. Our in vitro experiments showed that IL‐1β promoted iron influx and lead to chondrocytes iron overload. Excess iron in turn accelerated IL‐1β induced matrix‐degrading enzymes MMPs expression. Recently, oxidative stress and mitochondrial dysfunction were reported to play pivotal roles in the progression of OA.^28^, ^29^ However, the role of mitochondrial dysfunction in iron overload‐induced chondrocytes apoptosis and matrix‐degrading enzyme expression remain to be elucidated. Iron overload is common in tissues of elderly people and has been implicated to be responsible for many diseases or pathological conditions. In iron overloaded status, the free Fe^2+^ is increased and could catalyze the formation of free radicals, which could induce mitochondrial damage and reactive oxygen species generation, resulting in cell damage and eventually cell death.^15^, ^30^ Mitochondria are major cellular organelles that generate ROS, but excessive ROS production could in turn induce oxidative stress and mitochondrial dysfunction.^31^ Therefore, we first examined whether there is a connection between iron overload and mitochondrial dysfunction. We found that FAC promoted chondrocytes ROS production and mitochondrial fission proteins expression in a dose‐dependent manner. Our results indicated that excess iron could lead to mitochondrial dysfunction and morphology destruction. Furthermore, ROS production was significantly reduced by direct ROS scavenging with NAC as well as indirect ROS reduction through decreases in iron accumulation by iron chelation with DFO. Mitochondrial membrane potential decrease was also reversed after treatment with NAC or DFO. Our results indicated that iron accumulation in chondrocytes promoted ROS generation and caused mitochondrial dysfunction.

Our in vitro experiments also showed that FAC promoted chondrocytes apoptosis and matrix‐degrading enzymes MMPs expression. While antioxidant NAC treatment partly reversed iron overload‐induced mitochondrial dysfunction and MMPs production, NAC exhibited a similar protective effect with DFO, an iron chelator that could reduce cellular iron content, indicating oxidative stress and mitochondrial dysfunction play key roles in iron overload‐induced matrix‐degrading enzymes expression.

In conclusion, our results indicated that pro‐inflammatory cytokines could disturb cellular iron homeostasis via upregulating iron influx protein, TfR1, downregulating iron efflux protein FPN. Excess iron in chondrocytes leads to oxidative stress via ROS overproduction and mitochondrial dysfunction. Oxidative stress then induces upregulation of the crucial effector matrix‐degrading enzymes, MMP3 and MMP13. Our findings indicate that antioxidant treatment or local depletion of iron using an iron chelator would be effective therapeutic approaches for the treatment of iron overload‐induced cartilage degeneration.

## CONFLICT OF INTEREST

The authors declare that there are no conflicts of interest.

## AUTHOR CONTRIBUTIONS

**Xingzhi Jing:** Project administration (lead). **Ting Du:** Investigation (equal); Methodology (equal). **Tao Li:** Formal analysis (equal). **Xiaoxia Yang:** Data curation (equal). **Guodong Wang:** Data curation (equal). **Xiaoyang Liu:** Investigation (equal). **Zhensong Jiang:** Formal analysis (equal). **Xingang Cui:** Funding acquisition (equal).

## Data Availability

The data that support the findings of this study are available from the corresponding author upon reasonable request.
